# Multifunctional rGO/Y_2_O_3_@hydroxyapatite bioceramics: structural, optical, and biomedical properties

**DOI:** 10.1039/d5ra08618c

**Published:** 2026-01-23

**Authors:** Elbadawy A. Kamoun, Asmaa Elawadly, Merna H. Emam, Shahira H. EL-Moslamy, Asmaa M. Elzayat, E. M. Abdelrazek, Mohammed Sallah, Jong Yeog Son, Ahmed I. Ali

**Affiliations:** a Department of Chemistry, College of Science, King Faisal University Al-Ahsa 31982 Saudi Arabia ekamoun@kfu.edu.sa badawykamoun@yahoo.com +201283320302; b Physics Department, Faculty of Science, Mansoura University Mansoura 35516 Egypt; c Nanotechnology Research Center (NTRC), The British University in Egypt El-Shorouk City, Suez Desert Road, P.O. Box 43 Cairo 11837 Egypt; d Bioprocess Development Department (BID), Genetic Engineering and Biotechnology Research Institute (GEBRI), City of Scientific Research and Technological Applications (SRTA-City) New Borg El-Arab City Alexandria 21934 Egypt; e Department of Physics, College of Sciences, University of Bisha P.O. Box 344 Bisha 61922 Saudi Arabia; f Department of Applied Physics and Institute of Natural Sciences, College of Applied Science, Kyung Hee University Suwon 446-701 Republic of Korea jyson@khu.ac.kr; g Basic Science Department, Faculty of Technology and Education, Capital University (formerly Helwan University) Saray-El Qoupa, El Sawah St. 11281 Cairo Egypt ahmed_ali_2010@techedu.helwan.edu.eg ahmedalikh2024@khu.ac.kr; h Department of Mechanical Engineering (Integrated Engineering Program), Kyung Hee University 1732 Deogyeong-daero Yongin Gyeonggi 17104 South Korea

## Abstract

Hydroxyapatite (HAp)/reduced graphene oxide (rGO)/yttrium oxide (Y_2_O_3_) composites were prepared by ethanol-assisted wet blending (colloidal dispersion), followed by drying and heat treatment, and systematically characterized. This study investigates the structural, optical, and functional enhancement in hydroxyapatite (HAp) after the incorporation of reduced graphene oxide (rGO) and yttrium oxide (Y_2_O_3_), aiming to develop multifunctional bioceramics for dual applications in bone regeneration. HAp/rGO/Y_2_O_3_ composites were prepared by ethanol-assisted wet blending (colloidal dispersion), followed by drying and heat treatment, and systematically characterized. XRD analysis confirmed the phase purity of the composites and revealed reduced crystallite sizes upon doping, while FT-IR spectroscopy validated the presence of functional groups linked with phosphate, hydroxyl, and rGO/Y_2_O_3_ interactions. SEM investigation displayed dense, homogeneous morphologies with reduced particle sizes (∼380 nm), which are favorable for biomedical integration. UV/visible spectroscopy showed a significant narrowing of the optical bandgap from 5.42 eV in pure HAp to 4.73 eV in HAp/rGO/Y_2_O_3_ composites. This bandgap reduction was attributed to the π-conjugation of rGO and defect states introduced by Y_2_O_3_, thereby enhancing light absorption and rendering the composites promising candidates for photothermal and oxidative cancer therapies. The combined effect of rGO's electron transport and Y_2_O_3_'s reactive oxygen species (ROS) generation potential is expected to induce synergistic anticancer activity while preserving osteoconductivity. The tested AA3 formula demonstrated a promising level of biofilm reduction rates against Gram-positive bacteria, *e.g. Streptococcus pneumoniae* (97.27% ± 5.36%), *Staphylococcus epidermidis* (95.89% ± 2.85%), *Staphylococcus aureus* (94.77% ± 1.24%), and *Bacillus cereus* (92.90% ± 0.95%). Additionally, its minimum inhibitory dosage (MIC) against Gram-positive bacteria ranged from 80 to 100 µg mL^−1^, with a bactericidal effect (MBC) at 120 µg mL^−1^. *In vitro* cytotoxicity studies on *Vero* cells demonstrated the safety of our tested materials and composites. The MTT assay showed that HAP, Y_2_O_3_, rGO and their different composites (AA1 and AA3-AA5) exhibited CC_50_ values above 100 µg mL^−1^. By contrast, the CC_50_ value of the AA2 composite was detected at 8.5 µg L^−1^. Consequently, our composites have the potential to be used in biomedical applications. Overall, our results suggest that HAp/rGO/Y_2_O_3_ nanocomposites exhibit improved physicochemical and optical properties and hold substantial promise as dual-functional materials for integrated bone healing and localized cancer treatment platforms.

## Abstract 2

Hydroxyapatite (HAp)/reduced graphene oxide (rGO)/yttrium oxide (Y_2_O_3_) composites were prepared by ethanol-assisted wet blending (colloidal dispersion), followed by drying and heat treatment, and systematically characterized.

## Graphical abstract

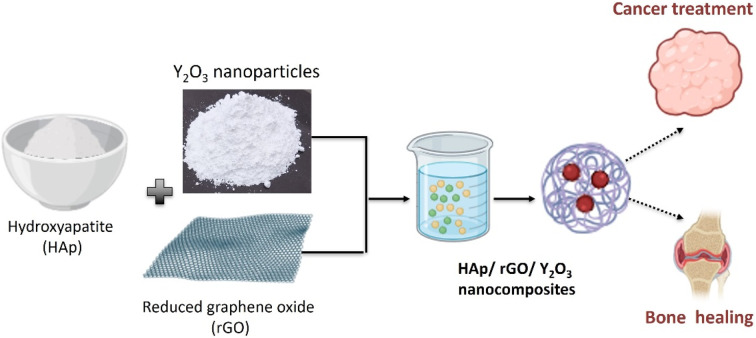

## 1 Introduction

Hydroxyapatite (HAp, Ca_10_(PO_4_)_6_(OH)_2_), a naturally occurring calcium phosphate bioceramic, is widely studied for its remarkable biocompatibility, bioactivity, osteoconductivity, and chemical resemblance to bone mineral.^1–3^ Owing to these properties, HAp has been broadly employed in orthopedic implants, bone tissue engineering, dental restorations, and biomedical fields, *e.g.* targeted drug carrier and cancer therapy.^4–6^ Its porous architecture, nontoxicity, and similarity to natural apatite render it an ideal scaffold for tissue regeneration. However, despite these advantages, the intrinsic brittleness, low mechanical strength, and limited functional versatility of pure HAp constrain its application,^4,7^ especially in load-bearing and oncological contexts.^1,8,9^ To overcome these limitations, significant research has focused on developing HAp-based nanocomposites by reinforcing it with functional nanomaterials.^4,10^ The inclusion of reinforcement agents aims to improve mechanical, electrical, and biological properties without compromising biocompatibility. While conventional dopants such as Al_2_O_3_, TiO_2_ and polyethylene enhance structural integrity, their bioinert nature often leads to reduced cellular affinity and biological performance.^4,7,10^ Consequently, attention has shifted toward bioactive nanomaterials such as reduced graphene oxide (rGO) and yttrium oxide (Y_2_O_3_), which offer multifunctionality with retained or improved biocompatibility.^4,11^

Reduced graphene oxide (rGO), a two-dimensional nanocarbon material, exhibits outstanding mechanical strength, electrical conductivity, high surface areas, and photothermal properties.^12,13^ Its sp^2^-hybridized carbon network and oxygen-containing functional groups facilitate interactions with biological macromolecules, improve the drug loading capacity, and offer platforms for photothermal cancer therapy.^14,15^ Furthermore, the high Young's modulus and flexibility of rGO improve the structural stability of composites, while its NIR absorbance enhances its utility in hyperthermia-assisted cancer treatment. The flat 2D morphology of rGO also allows intimate contact with cells, leading to increased uptake and enhanced bioresponse, but its safety profile demands further validation.^16,17^

Yttrium oxide (Y_2_O_3_), a rare-earth metal oxide known for its thermal and chemical stability, has emerged as a promising additive in biomedical composites. Y_2_O_3_ nanoparticles possess unique characteristics such as antimicrobial activity, oxidative stress induction, and radio luminescent behavior. In oncology, Y_2_O_3_ has demonstrated the ability to generate reactive oxygen species (ROS), thereby inducing cancer cell apoptosis. Its incorporation into polymeric or ceramic matrices has also been shown to improve thermal resistance and mechanical reinforcement. Furthermore, Y_2_O_3_'s white color and radiodensity make it suitable for imaging-guided therapies and theragnostics.^18–20^

The synergistic integration of HAp, rGO, and Y_2_O_3_ is expected to afford a multifunctional nanocomposite that not only retains the osteoconductive and bioactive nature of HAp but also gains enhanced structural integrity from rGO and therapeutic efficacy from Y_2_O_3_. Such a composite can serve dual roles: structural regeneration of bone tissue and targeted cancer intervention *via* photothermal and oxidative pathways. Despite the promising outlook, limited studies have systematically investigated the combined effect of rGO and Y_2_O_3_ on the physicochemical and biological performance of HAp in cancer-related applications.

The present work aims to synthesize HAp/rGO/Y_2_O_3_ nanocomposites *via* a sol–gel method and to evaluate their structural, morphological, optical, and biological characteristics. Techniques such as XRD, Raman spectroscopy, zeta size analysis, UV/visible spectroscopy, and FTIR spectroscopy are employed to examine compositional integrity and particle interactions. In parallel, antimicrobial activity and *in vitro* cell culture assessments are conducted to validate the composite's biomedical potential. Through this integrated approach, this study seeks to advance the development of hybrid HAp-based platforms for next-generation cancer therapy and regenerative medicine.

## 2 Materials and methods

### 2.1. Materials

Calcium chloride dihydrate (CaCl_2_·2H_2_O; 99.9%), diammonium hydrogen phosphate ((NH_4_)_2_HPO_4_; 99.9%), ammonium hydroxide (NH_4_OH; 99.7%), graphite powder, potassium permanganate (KMnO_4_; 99.9%), concentrated sulfuric acid (H_2_SO_4_; 99.8%), hydrogen peroxide (H_2_O_2_), and hydrochloric acid (HCl) were purchased from Sigma-Aldrich, Germany. Yttrium oxide (Y_2_O_3_; 99.99%) was purchased from Sigma-Aldrich, Korea. Deionized water (DIW) was used throughout the experimental procedures.

The *Vero* cell line (American Tissue Culture Collection, ATCC) was obtained from the Vaccination and Sera Collection Organization (VACSERA), Egypt. Dulbecco's modified Eagle medium (DMEM), fetal bovine serum (FBS), and trypsin–EDTA (0.25%, phenol red) were purchased from Gibco, USA. Sodium pyruvate (100 mM), l-glutamine (200 mM), and penicillin/streptomycin (100 IU mL^−1^) were purchased from Lonza, Belgium. The (3-[4,5-dimethylthiazol-2-yl]-2,5-diphenyltetrazolium bromide (MTT) dye and dimethyl sulfoxide (DMSO, research grade) were purchased from Serva Electrophoresis GmbH, Germany.

### 2.2. Synthesis of hydroxyapatite (HAp)

Hydroxyapatite (HAp) nanoparticles were synthesized *via* a conventional wet-chemical precipitation method. A 0.5 M solution of CaCl_2_·2H_2_O and a 0.3 M solution of (NH_4_)_2_HPO_4_ were prepared separately in 100 mL of DIW. The calcium precursor was added dropwise into the phosphate solution under continuous magnetic stirring at 1200 rpm while maintaining the pH at 11 ± 0.1 using a dilute NH_4_OH solution. The resulting mixture was stirred for an additional 2 h to ensure complete precipitation. The suspension was then aged for 24 h under ambient conditions to promote crystallization. The precipitate was collected by filtration, washed carefully with DIW to remove residual ions, and dried at 50–60 °C. The resulting HAp powder was ground well and stored for further use.

### 2.3. Synthesis of reduced graphene oxide (rGO)

Graphene oxide (GO) was synthesized using a modified Hummers' method. Briefly, 2.0 g of graphite flakes and 2.0 g of NaNO_3_ were added to 46 mL of concentrated H_2_SO_4_ and stirred for 2 h. The reaction mixture was maintained at 0 °C using an ice bath. Subsequently, 3.0 g of KMnO_4_ was added slowly under continuous stirring while keeping the temperature below 20 °C to control the oxidation process. During this step, the suspension color changed from black to brown. The temperature was then increased to 35 °C, and stirring was continued for 30 min. Next, 92 mL of distilled water was added slowly (exothermic step), followed by the addition of 150 mL of distilled water in one portion. After stirring for 1 min, 20 mL of H_2_O_2_ was added to terminate the reaction, resulting in a color change from brown to greenish-yellow. The obtained suspension was ultrasonicated and washed several times with distilled water, 5% HCl, and acetone. Finally, the product was dried in a hot-air oven at 350 °C for 2 h and collected for subsequent use. This thermal treatment yielded the reduced graphene oxide (rGO) powder.

### 2.4. Synthesis of yttrium oxide (Y_2_O_3_) nanoparticles

Yttrium oxide nanoparticles were prepared *via* a precipitation-calcination route. An aqueous solution of Y_2_O_3_ was prepared and neutralized with dropwise addition of ammonium hydroxide under stirring. The reaction mixture was stirred for 30 min to ensure the complete precipitation of yttrium hydroxide. The resulting precipitate was washed repeatedly with DIW, followed by drying at 80 °C for 24 hours. The dried product was then calcined at 800 °C for 180 minutes to obtain the white Y_2_O_3_ nanopowder.

### 2.5. Fabrication of HAp/Y_2_O_3_/rGO nanocomposites

**Fig. 1:**
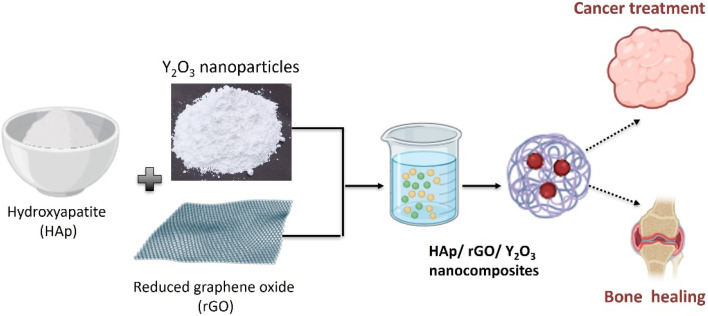
Schematic of the ethanol-assisted wet blending (colloidal dispersion) and thermal treatment steps used to prepare the HAp/rGO/Y_2_O_3_ nanocomposites for dual applications.

The nanocomposite samples were prepared by ethanol-assisted wet blending (Fig. 1), and their compositions are summarized in Table 1. Hydroxyapatite (HAp), reduced graphene oxide (rGO), and yttrium oxide (Y_2_O_3_) were weighed according to the target ratios and ground separately to enhance homogeneity. The powders were then mixed and dispersed in ethanol under magnetic stirring to form a uniform colloidal suspension (particle sol), where ethanol served as a dispersion medium rather than a solvent for the solids. After solvent removal by drying, the obtained dried mass was ground and heat-treated to promote consolidation/crystallization, yielding the final HAp/rGO/Y_2_O_3_ nanocomposite powders, which were stored for subsequent characterization.

**Table 1:** Detailed description of the fabricated nanocomposite samples in terms of their codes, ratios and compositions

Sample code	HAP	rGO	Y_2_O_3_	Sample contents
AA1 (S1)	98	0	2	98 wt% HAP/2 wt% Y_2_O_3_
AA2 (S2)	98	0.5	1.5	98 wt% HAP/0.5 wt% rGO/1.5 wt% Y_2_O_3_
AA3 (S3)	98	1	1	98 wt% HAP/1 wt% rGO/1 wt% Y_2_O_3_
AA4 (S4)	98	1.5	0.5	98 wt% HAP/1.5 wt% rGO/0.5 wt% Y_2_O_3_
AA5 (S5)	98	2	0	98 wt% HAP/2 wt% rGO

### 2.6. Antimicrobial effectiveness

In this work, two antimicrobial methods were applied to test the antimicrobial efficacy of the evaluated nanoformulations (AA1, AA2, AA3, AA4, and AA5), namely, agar-well diffusion approach and microdilution strategy.

#### 2.6.1 The agar-well diffusion method

The microbiological experiment employed the agar diffusion technique.^21^ The following multidrug-resistant (MDR) human pathogens were used to test the nanoformulations' antimicrobial efficacy: *Staphylococcus epidermidis* (ATCC14990), *Staphylococcus aureus* (ATCC25923), *Streptococcus pneumoniae* (ATCC33400), *Bacillus cereus* (ATCC19637), *Candida albicans* (ATCC10231), *Candida krusei* (ATCC6258), *Candida tropicalis* (ATCC13803), *Candida glabrata* (ATCC66032), *Escherichia coli* (ATCC10536), *Klebsiella pneumoniae* (ATCC10031), *Pseudomonas aeruginosa* (ATCC27853), and *Salmonella paratyphi* (ATCC9150). These MDR human pathogens were collected from GEBRI, SRTA-City, Alexandria, Egypt. To generate microbial suspension cultures, the signal colony of each pathogen was subcultured in 10 mL of a nutrient broth medium (0.5% NaCl, 0.5% peptone, 0.2% beef extract, and 0.3% yeast extract) and then incubated at 200 rpm and 37 °C. Afterward, the generated cells were collected and centrifuged for 10 minutes at 12 000 rpm. These pellets were carefully resuspended in a 0.85% NaCl saline solution to achieve a microbial suspension with 1 × 10^6^ CFU mL^−1^. After the prepared nutritious agar medium was cooked at 121 °C for 25 minutes, 20 mL of a sterile agar medium was poured into *Petri* plates. 100 µL of the microbial suspension was uniformly spread on the agar surfaces using a cotton swab stick. 100 µg of each formula was stirred with 1 mL of distilled water to yield nanoformula suspensions, which were then sonicated for 10 minutes before being used. After that, sterile cork borers (6 mm in diameter) were used to drill wells, which were filled with 100 µL of each individually sonicated nanoformula. The wells were then left undisturbed for 30 minutes. The subsequent 24 h were spent keeping these plates at 37 °C. The diameter of the millimeter-sized zone of inhibition that appeared around the well after this incubation period was measured to determine the antimicrobial efficacy.^22^

#### 2.6.2 Turbidimetric assay

The antimicrobial efficiency of the examined nanoformulations against MDR human pathogens was determined by measuring the produced turbidity. The evaluated nanoformulations in the nutrient broth at a concentration of 100 µg mL^−1^ were inoculated with the chosen MDR human pathogens. As a control, a nutrient broth without any of the evaluated nanoformulations was prepared.^23^ To determine the sensitivity of the tested human pathogens in this experiment, a broth mixture was cultured in conical flasks at 37 °C with a continuous shaker at 200 rpm for 24 hours. The optical density (OD) at 600 nm was then determined using a spectrophotometer. The percentages of microbial growth inhibition were then calculated by the formula shown below:^24^1
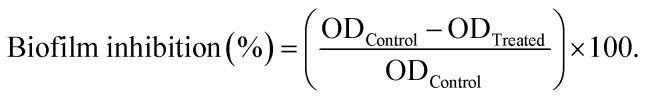


#### 2.6.3 Estimation of minimum inhibitory concentrations (MICs) and minimal bactericidal/fungicidal concentrations (MBCs/MFCs)

In these experiments, the modified microdilution method was used to determine the MIC and MBC/MFC of our nanoformulation suspensions against tested human pathogens. 1 mL of each of the following nanoformula doses was added to 24-hours-old bacterial cultures (1 × 10^7^ CFU mL^−1^): 20, 40, 60, 80, 100, 120, 140, 160, 180, 200, and 220 µg mL^−1^. After that, the mixture was incubated at 37 °C for 24 h with shaking at 200 rpm. As a control, a medium without any of the tested nanoformulations was prepared. The MIC was determined by rating the OD of turbidity at a wavelength of 600 nm. In our study, the MIC values were determined to reflect the nanoformulation's lowest dose, at which more than 95% of microbial growth was inhibited.^25^ 50 µL of the broth was taken from all tested dosages (MIC and higher doses) that showed no sign of growth or turbidity and streaked over nutrient agar plates to detect the MBC/MFC. Plates were then incubated for an additional 24 h at 37 °C. The MBC and MFC were defined as the lowest dosages of the nanoformula that prevented pathogen growth on free nanoformula media.

#### 2.6.4 Statistical analysis

Three replicates of each experiment were performed twice. As the model for all outcomes, the mean and standard deviation (M ± SD) were implemented. Minitab 18 was utilized for the one-way analysis of variance (*P*-value ≤ 0.05) in this study. Tukey's results were considered significant when the variance levels were less than 0.05.

### 2.7. Cell culture tests

*Vero* cells were subcultured into tissue culture flasks and maintained in Dulbecco's modified Eagle's medium (DMEM) supplemented with 10% fetal bovine serum (FBS), 1% penicillin/streptomycin, 1% sodium pyruvate, and 1% l-glutamine. The cells were incubated under standard conditions, 5% CO_2_ and 37 °C. The toxicity of the tested samples was investigated on *Vero* cells by performing the 3-[4,5-dimethylthiazol-2-yl]-2,5-diphenyltetrazolium bromide (MTT) assay. The cells were seeded in a 96-well tissue culture plate at a density of 10 × 10^3^ cells per well. Before applying the materials, they were sterilized with ultraviolet radiation. The cells were treated with 2-fold serial dilutions of the tested materials starting from 100 to 12.5 µg mL^−1^. The cells were treated and incubated under standard conditions for 48 h. Afterwards, 100 µL of the MTT dye was added to each well, and the plate was incubated for 3.5 h. Then, the dye was discarded, and 30 µL of DMSO was added to each well to solubilize the formed formazan crystals. The plate was placed in a microplate reader (CLARIOstar, BMG Labtech, Germany) for reading the absorbance.^26^ The cytotoxic concentrations (CC_50_, the highest dilution of the tested sample that causes 50% reduction in the cellular viability) of the investigated materials were determined using the GraphPad Prism software v.8.0.2. The cellular viability (%) was calculated according to the following equation:^27^2
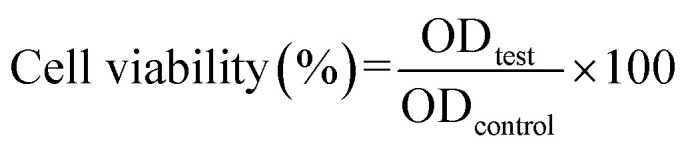
where OD test is the mean optical density value of the tested sample and OD control is the mean optical density value of the control sample.^26^

## 3 Results and discussion

### 3.1. XRD patterns

**Fig. 2:**
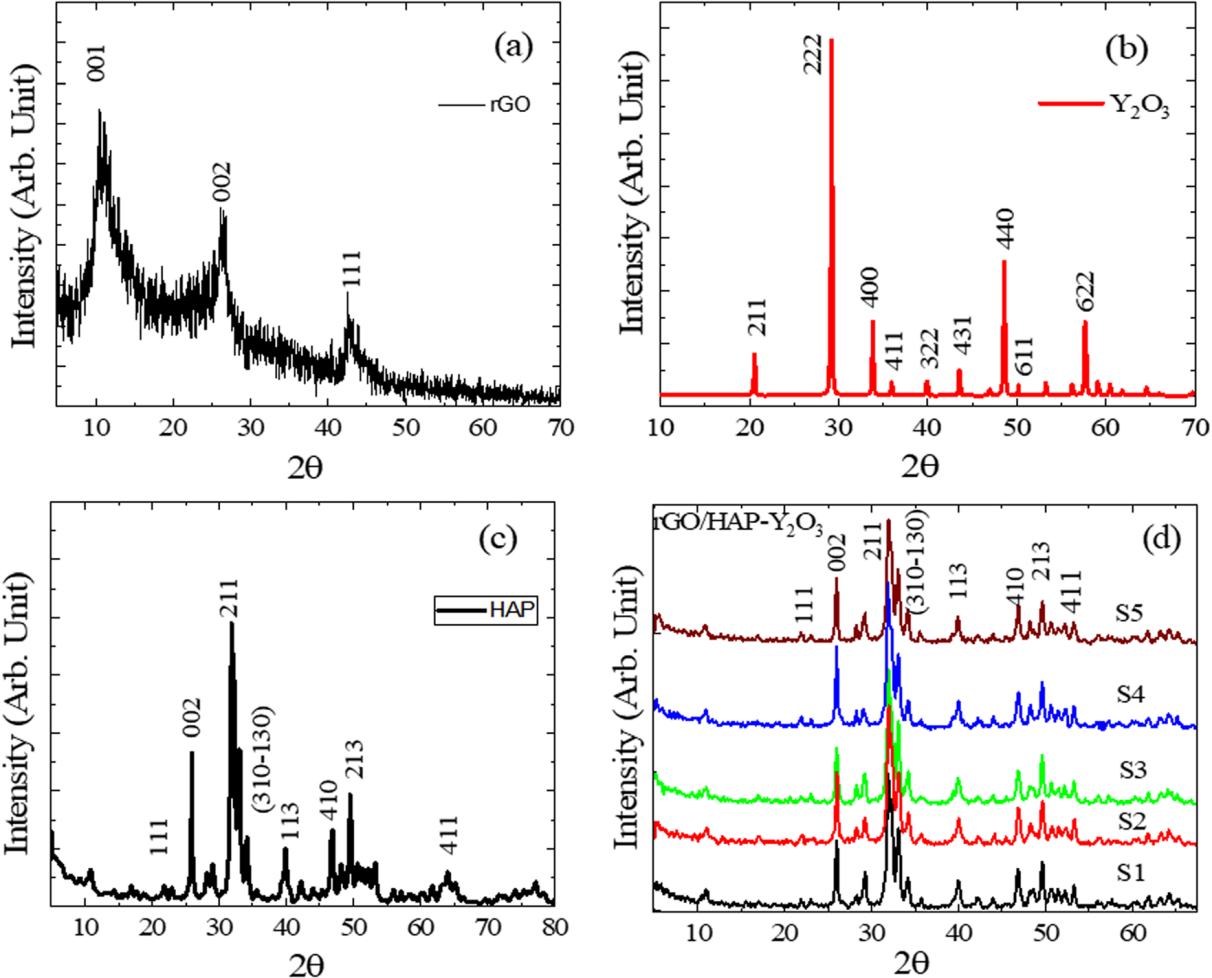
XRD patterns of the raw and composite materials: (a) rGO showing broad peaks corresponding to a disordered carbon structure; (b) Y_2_O_3_ exhibits sharp peaks indicative of its cubic crystalline phase; (c) HAp revealing well-defined reflections consistent with the hexagonal Ca_10_(PO_4_)_6_(OH)_2_ phase; and (d) HAp/rGO/Y_2_O_3_ composite samples, confirming the coexistence of HAp, Y_2_O_3_, and rGO phases without the formation of undesirable secondary phases and illustrating peak broadening due to lattice distortion and nanostructuring effects.

The XRD pattern of reduced graphene oxide (rGO), prepared by the modified Hummers' oxidation method, followed by thermal treatment at 350 °C for 2 h (Section 2.3), exhibits broad diffraction features centered at 2*θ* ≈ 11.1°, 21.2°, and 42.4° (Fig. 2a). The weak/broad reflection at ∼11.1° can be attributed to residual GO-like (001) stacking, indicating the incomplete removal of oxygen functionalities, whereas the broad band around 21.2° is assigned to the (002) reflection of turbostratic/disordered graphitic carbon. The feature at ∼42.4° is commonly associated with the (100) plane of disordered carbon. The broadness of these reflections confirms the poorly ordered (turbostratic) structure typical of thermally reduced GO. In Fig. 2b, the crystalline nature of Y_2_O_3_ is clearly evidenced by a series of sharp, high-intensity peaks. The major reflections are indexed to the cubic bixbyite structure of Y_2_O_3_, with prominent peaks observed at 2*θ* ≈ 29.1° (222), 33.2° (400), 48.1° (440), and 57.6° (622), among others. These reflections match well with the standard (JCPDS card no. 41-1105), indicating phase-pure Y_2_O_3_ with no secondary phases. The sharpness and intensity of the peaks also confirm its high degree of crystallinity. Fig. 2c shows the XRD pattern of hydroxyapatite, in which all diffraction peaks can be assigned to the hexagonal phase of Ca_10_(PO_4_)_6_(OH)_2_, consistent with JCPDS card no. 09-0432. Major diffraction peaks are located at 2*θ* ≈ 25.9° (002), 31.8° (211), 32.2° (112), 32.9° (300), and 39.8° (310), confirming the formation of phase-pure HAp. The presence of well-resolved peaks with moderate sharpness also indicates crystalline hydroxyapatite. In addition, XRD results confirm the successful synthesis and phase purity of the three key starting components, rGO, Y_2_O_3_, and HAp, used for the fabrication of HAp/rGO/Y_2_O_3_ nanocomposites. These patterns serve as reference baselines for evaluating phase evolution, crystallinity, and structural integrity in the composite systems.^21,22^

### 3.2. XRD analysis of HAp/rGO/Y_2_O_3_ composites

Fig. 2d illustrates the XRD patterns of hydroxyapatite-based nanocomposites incorporating rGO and Y_2_O_3_ prepared *via* the sol–gel method. This analysis was used to evaluate crystallographic behavior, phase compositions, and structural interactions resulting from the incorporation of rGO and Y_2_O_3_ into the HAp matrix. Across all composite samples, the characteristic diffraction peaks of HAp are preserved, particularly those at 2*θ* ≈ 25.9° (002), 31.8° (211), 32.9° (300), and 39.8° (310), corresponding to the hexagonal Ca_10_(PO_4_)_6_(OH)_2_ phase (JCPDS card no. 09-0432). The persistence of these peaks confirms that the HAp phase remains structurally stable upon the incorporation of both dopants, and no decomposition into secondary calcium phosphate phases is observed. In addition to HAp peaks, diffraction signals corresponding to Y_2_O_3_ are identified at 2*θ* ≈ 29.1° (222), 33.2° (400), and 48.1° (440), which agree with the cubic phase of Y_2_O_3_ (JCPDS card no. 41-1105). These peaks confirm the successful inclusion of crystalline Y_2_O_3_ into the HAp matrix without any chemical reaction or phase transformation.

The presence of rGO is suggested by a low-intensity broad peak appearing at 2*θ* ≈ 11–12°, corresponding to the (001) reflection of graphene oxide. Although rGO is often weakly crystalline, its detection *via* XRD can be limited due to its partial amorphous structure and low concentration. However, the slight broadening of the baseline and the attenuation of HAp peak intensities in the composite may indicate the homogeneous dispersion of rGO nanosheets within the ceramic matrix. A comparison between samples indicates a slight shift and broadening of HAp peaks with increasing rGO and Y_2_O_3_ contents, which can be attributed to lattice distortion, strain effects, and nanocrystallinity introduced by the dopants. These modifications suggest successful integration at the nanoscale, potentially improving the composite's mechanical and functional properties. In summary, XRD data confirms the coexistence of HAp, rGO, and Y_2_O_3_ phases in the synthesized composites without the formation of undesirable secondary phases. The retention of crystallinity, along with peak broadening, implies enhanced dispersion and potential interfacial interactions, making these composites promising candidates for biomedical applications, particularly in bone regeneration and targeted cancer therapy.

### 3.3. FT-IR spectroscopic analysis

**Fig. 3:**
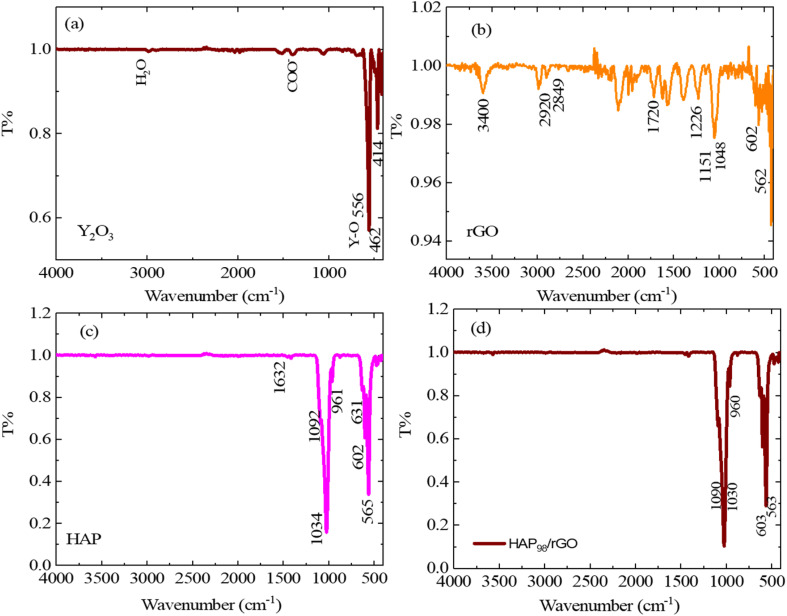
FT-IR spectra (4000–400 cm^−1^) of the starting materials: (a) Y_2_O_3_, (b) rGO, (c) HAp, and (d) HAp/rGO.

**Fig. 4:**
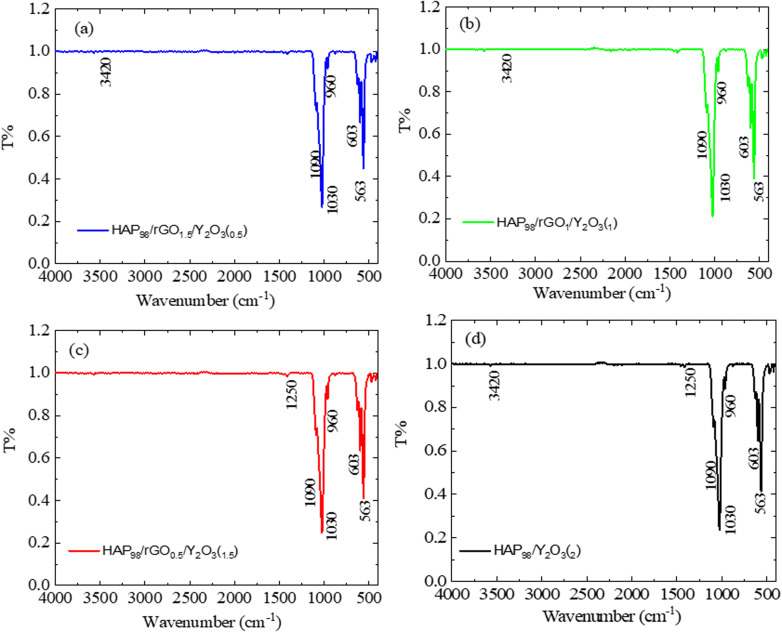
FT-IR spectra (4000–400 cm^−1^) of the HAp/rGO/Y_2_O_3_ nanocomposites: (a) HAP_98_/1.5 wt% rGO/0.5 wt% Y_2_O_3_, (b) HAP_98_/1.0 wt% rGO/1.0 wt% Y_2_O_3_, (c) HAP_98_/0.5 wt% rGO/1.5 wt% Y_2_O_3_, and (d) HAP_98_/2 wt% Y_2_O_3_.

The chemical structures and surface functional groups of the starting powders and prepared composites were examined by FT-IR spectroscopy (Fig. 3 and 4). Fig. 3 shows the FT-IR spectra of the starting materials, Y_2_O_3_, rGO, and HAp, and the binary HAp/rGO composite. For Y_2_O_3_ (Fig. 3a), the characteristic lattice vibrations of Y–O appear in the low-wavenumber region, with distinct absorption bands at approximately 556 cm^−1^ and 462 cm^−1^, confirming the oxide framework. Weak features attributed to H_2_O and CO_2_ are also observed and are commonly associated with adsorbed moisture and atmospheric CO_2_, respectively. The spectrum of rGO (Fig. 3b) exhibits a broad band centered at ∼3400 cm^−1^, which is attributable to O–H stretching from residual hydroxyl groups and/or adsorbed water. The bands at ∼2929 cm^−1^ and 2849 cm^−1^ correspond to C–H stretching vibrations. The peak near 1720 cm^−1^ is assigned to C=O stretching (carboxyl/carbonyl groups), while the absorptions around 1226 cm^−1^ and 1151 cm^−1^ are attributed to C–O/C–O–C stretching of epoxy/ether-type functionalities, indicating that oxygen-containing groups remain on the rGO surface. For HAp (Fig. 3c), the phosphate vibrational modes are clearly observed: the bands at ∼563 cm^−1^ and 602 cm^−1^ correspond to the *ν*_4_ bending modes of PO_4_^3−^, the band at ∼961 cm^−1^ is assigned to the *ν*_1_ symmetric stretching mode, and the bands at ∼1034 cm^−1^ and 1092 cm^−1^ correspond to the *ν*_3_ asymmetric stretching modes. In addition, the band near 1632 cm^−1^ is associated with the H–O–H bending of adsorbed water. In the HAp/rGO composite (Fig. 3d), the characteristic phosphate bands of HAp (notably in the 560–602 cm^−1^ and 1030–1090 cm^−1^ regions) are retained, indicating the preservation of HAp's chemical structure after mixing with rGO. The rGO-related bands are comparatively weaker/less distinct in the composite spectrum, which can be attributed to the lower rGO content and their overlap with the phosphate-rich fingerprint region. Overall, Fig. 3 establishes the reference functional group signatures of each starting material and confirms the successful formation of the HAp/rGO composite, while the ternary composite features are discussed in Fig. 4.

### 3.4. FT-IR spectroscopic analysis of HAp/rGO/Y_2_O_3_ composites

The FT-IR spectra of the synthesized HAp/rGO/Y_2_O_3_ composite powders are presented in Fig. 4. The spectra retain the characteristic vibrational features of hydroxyapatite and the signals related to the graphene-derived phase and yttrium oxide, supporting the successful formation of the ternary system. The apatite phase is confirmed by the prominent phosphate bands: the absorptions at ∼563 and ∼602 cm^−1^ correspond to the *ν*_4_ bending modes of PO_4_^3−^, while the band at ∼960–961 cm^−1^ is assigned to the *ν*_1_ symmetric stretching vibration. The bands in the 1030–1090 cm^−1^ region (often observed at 1030–1034 and 1090–1092 cm^−1^) are attributed to the *ν*_3_ asymmetric stretching of PO_4_^3−^ units, indicating that the HAp phosphate framework is preserved in the composites. The broad absorption band centered at 3400–3420 cm^−1^ is associated with O–H stretching (structural –OH and/or adsorbed moisture), and the band at 1630–1632 cm^−1^ corresponds to the H–O–H bending of adsorbed water, which is commonly observed for HAp-based powders. Contributions from the graphene-derived component are evidenced by the band at ∼1720 cm^−1^, attributed to C=O stretching (carboxyl/carbonyl groups), and the bands in the 1220–1250 cm^−1^ region (often appearing at ∼1226 cm^−1^), which can be assigned to the C–O/C–O–C stretching of residual oxygen-containing functionalities. These features indicate that the carbon phase retains oxygenated groups that can promote interfacial interactions with the ceramic phases. The incorporation of Y_2_O_3_ is supported by the appearance of Y–O lattice vibrations in the low-wavenumber region (typically observed in the 400–600 cm^−1^ range), which may partially overlap with the phosphate bending bands of HAp. Overall, the combined presence of phosphate (HAp), carbon–oxygen functionalities (rGO), and Y–O vibrations (Y_2_O_3_), together with the absence of new unexpected absorption bands, confirms the chemical compatibility of the constituents and the successful preparation of HAp/rGO/Y_2_O_3_ ternary composite powders.

### 3.5. SEM morphology of the synthesized composites

**Fig. 5:**
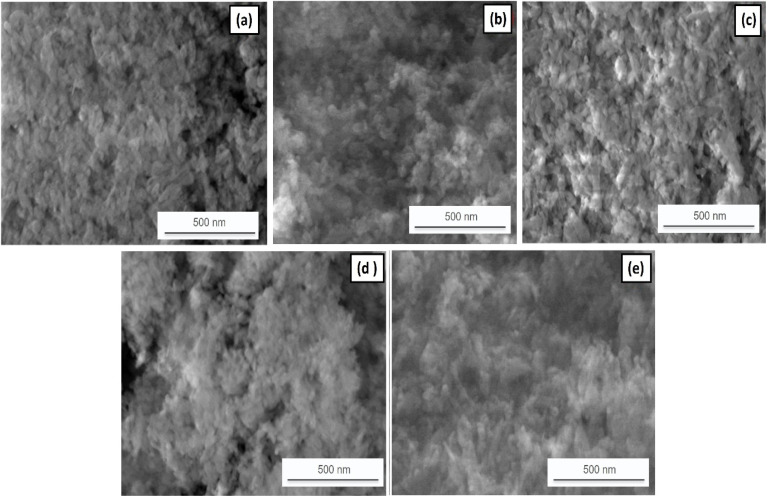
SEM micrographs of the fabricated samples: (a) AA1: 98 wt% HAp/2 wt% Y_2_O_3_; (b) AA2: 98 wt% HAp/0.5 wt% rGO/1.5 wt% Y_2_O_3_; (c) AA3: 98 wt% HAp/1 wt% rGO/1 wt% Y_2_O_3_; (d) AA4: 98 wt% HAp/1.5 wt% rGO/0.5 wt% Y_2_O_3_; and (e) AA5: 98 wt% HAp/2 wt% rGO. The micrographs show quasi-spherical granular features with noticeable agglomeration typical of dried powders (scale bar = 500 nm).

The morphology of the synthesized HAp/rGO/Y_2_O_3_ nanocomposite powders was investigated using SEM (Fig. 5). The micrographs show a generally homogeneous granular texture with quasi-spherical features and visible interparticle boundaries, consistent with polycrystalline clusters formed during wet blending, followed by thermal treatment. Notably, the powders exhibit pronounced agglomeration, and most features observed correspond to secondary aggregates rather than isolated primary nanoparticles. This behavior is expected for dried nanocomposite powders because solvent evaporation, capillary forces during drying, and strong interparticle attractions can promote aggregation. Accordingly, the particle dimensions obtained from SEM were determined by digital image analysis (scale-calibrated measurements of clearly resolved quasi-spherical features) and are reported as the apparent aggregate size rather than a true dispersion size distribution. To support particle size evaluation in suspension, the DLS/zetasizer particle size distribution data for AA1–AA5 are provided and cited in SI Fig. S1–S5. Taken together, SEM confirms the agglomerated yet compositionally consistent morphology of the nanocomposite powders, while the dispersion-based particle size distribution is best represented by DLS.

### 3.6. Optical properties

**Fig. 6:**
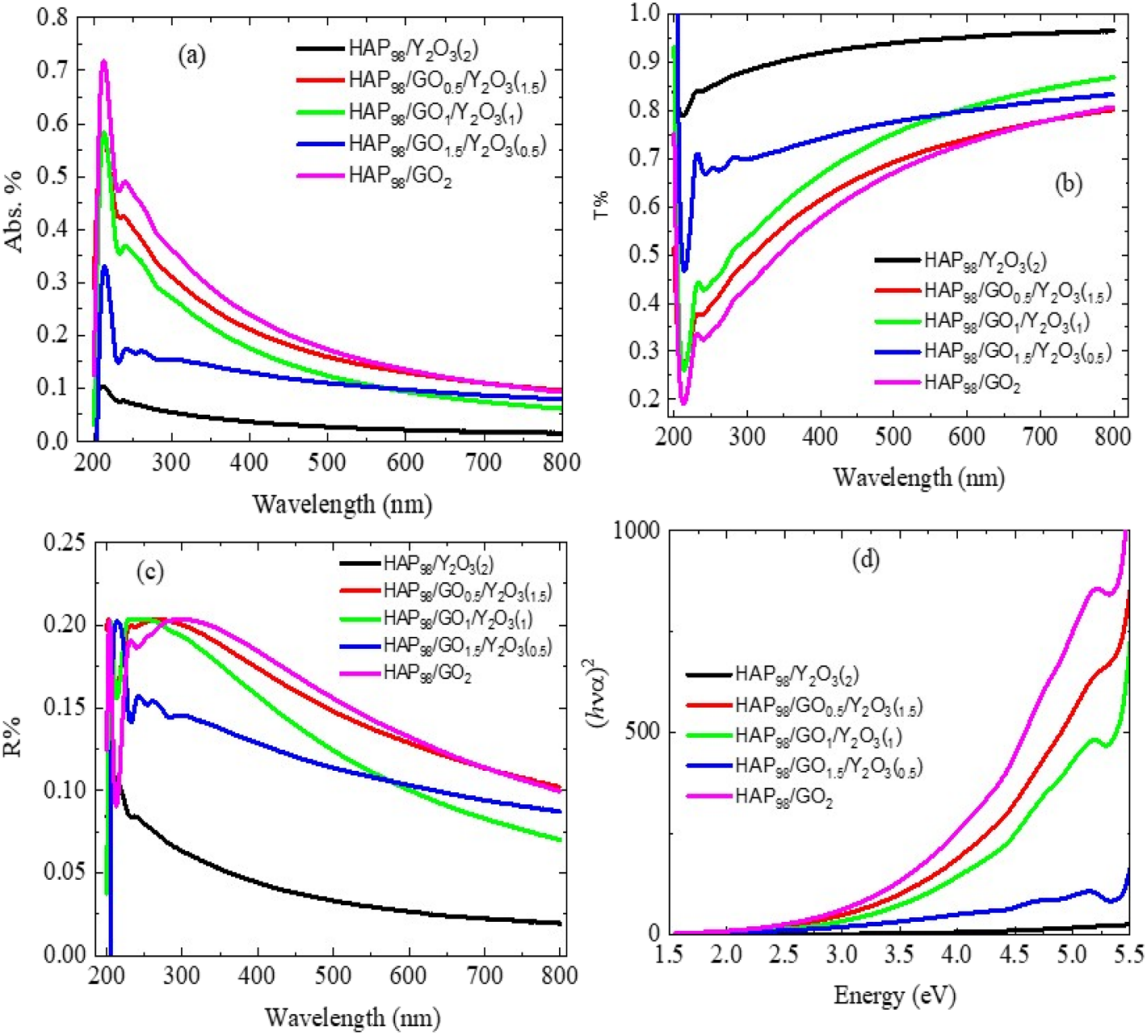
UV/vis optical spectra of the prepared powders showing the (a) absorbance (Abs.%), (b) transmittance (*T*%), (c) reflectance (*R*%), and (d) Tauc plots ((*αhν*)^2^*versus* photon energy (eV)) for optical band-gap estimation. The curves correspond to the compositions AA1 (98 wt% HAp/2 wt% Y_2_O_3_), AA2 (98 wt% HAp/0.5 wt% rGO/1.5 wt% Y_2_O_3_), AA3 (98 wt% HAp/1.0 wt% rGO/1.0 wt% Y_2_O_3_), AA4 (98 wt% HAp/1.5 wt% rGO/0.5 wt% Y_2_O_3_), and AA5 (98 wt% HAp/2 wt% rGO).

The optical behavior of the synthesized HAp/rGO/Y_2_O_3_ nanocomposites was investigated using UV/visible spectroscopy, and the corresponding absorbance spectra are presented in Fig. 6. All samples exhibit a strong absorption band in the ultraviolet (UV) region (typically 200–400 nm), characteristic of the phosphate groups in HAp. This intrinsic absorption is primarily associated with electronic transitions within PO_4_^3−^ units. Notably, a red shift in the absorption edge is observed upon the incorporation of rGO into the HAp matrix. This shift indicates a narrowing of the optical bandgap, which is attributed to the presence of π-conjugated carbon domains in rGO that introduce mid-gap states and facilitate charge transfer interactions with the HAp framework. The extended π-electron network in rGO enhances the photon absorption capability of the composite, which is beneficial for applications involving photothermal or photodynamic therapy. The addition of Y_2_O_3_ further modifies the absorption profile. While Y_2_O_3_ has a relatively wide bandgap (>5 eV), its incorporation contributes to defect-level formation, particularly oxygen vacancies and interstitials, which in turn affect the absorption intensity and slope of the edge. The slight broadening of the absorption tail in the composites' spectra supports the hypothesis of defect-mediated transitions, which enhance sub-bandgap absorption. Furthermore, the synergistic interaction between HAp, rGO, and Y_2_O_3_ enhances light-harvesting efficiency across a broader spectral window. This tunability is especially significant for biomedical applications, where materials are expected to absorb in the near-infrared (NIR) region to facilitate photothermal conversion for cancer treatment. Although the absorption onset remains in the UV range, the presence of rGO provides an opportunity to engineer the band structure toward visible and NIR responsiveness through compositional and structural control. Finally, the UV/vis spectra confirm that the incorporation of rGO and Y_2_O_3_ into HAp not only modifies the optical band structure but also introduces defect states and electronic coupling, which are essential for multifunctional nanobiomedical applications.

### 3.7. Optical bandgap analysis

**Fig. 7:**
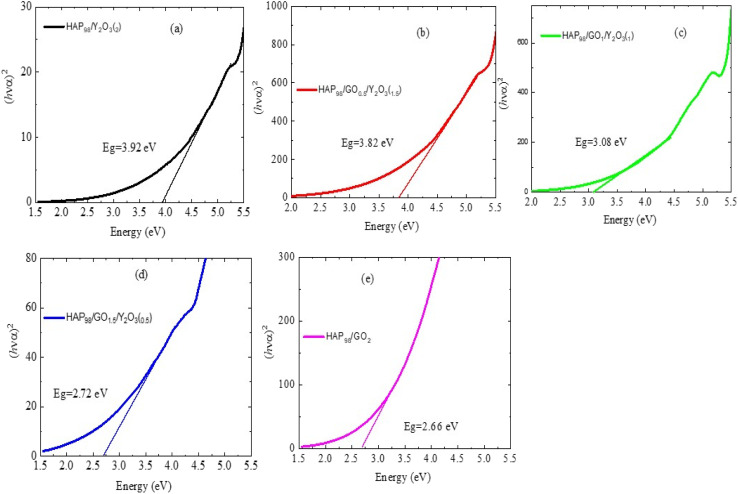
Tauc plots ((*αhν*)^2^*versus* photon energy (eV)) for estimating the optical band gap (*E*_g_) of the prepared samples: (a) AA1 (98 wt% HAp/2 wt% Y_2_O_3_), (b) AA2 (98 wt% HAp/0.5 wt% rGO/1.5 wt% Y_2_O_3_), (c) AA3 (98 wt% HAp/1.0 wt% rGO/1.0 wt% Y_2_O_3_), (d) AA4 (98 wt% HAp/1.5 wt% rGO/0.5 wt% Y_2_O_3_), and (e) AA5 (98 wt% HAp/2 wt% rGO). The extracted bandgap values are 3.92 eV, 3.82 eV, 3.08 eV, 2.72 eV, and 2.66 eV, respectively, showing a progressive decrease in *E*_g_ on increasing the rGO content.

Fig. 7 shows the *Tauc* plots used to estimate the optical bandgap (*E*_g_) values of pure HAp and its composites modified with rGO and Y_2_O_3_. The (*αhν*)^2^*vs. hν* plots were constructed under the assumption of a direct allowed transition, which is typical for hydroxyapatite-based materials. For the pure HAp sample, the extrapolated bandgap is approximately 5.42 eV, which aligns with the expected wide bandgap of stoichiometric HAp.^23,24^ Upon the introduction of rGO, a notable decrease in the bandgap is observed. For the HAp/rGO composites, the bandgap reduces (3.92 eV, 3.82 eV, 3.08 eV, 2.72 eV, and 2.66 eV) (Table 2), indicating enhanced sub-bandgap absorption. This reduction is attributed to the delocalized π-electrons of rGO, which introduce mid-gap states and facilitate photon-induced electronic transitions at lower energies. Further, the incorporation of Y_2_O_3_ into the HAp/rGO matrix continues to modulate the bandgap values. The ternary composite (HAp/rGO/Y_2_O_3_) shows a slightly lower bandgap of approximately 2.66 eV, reflecting the combined influence of defect states, lattice distortion, and interfacial interactions on the ceramic and carbonaceous components. Y_2_O_3_ likely contributes additional localized energy states *via* oxygen vacancies or interstitial yttrium, enhancing the material's photon absorption capability in a broader energy range. This gradual decrease in the bandgap with compositional variation confirms the synergistic roles of rGO and Y_2_O_3_ in tailoring the electronic structure of hydroxyapatite. Such a tuning of optical properties is particularly significant for biomedical applications, where absorption characteristics must align with therapeutic windows in photothermal or photodynamic cancer treatment.

**Table 2:** The optical bandgap (*E*_g_) values for all composites

Sample	Energy gap (eV)
S1	3.92
S2	3.82
S3	3.08
S4	2.72
S5	2.66

### 3.8. Zetasizer measurements of composites

The particle size and zeta size analyses of the fabricated composites, AA1, AA2, AA2, AA4 and AA5 (as displayed in Table 1), are displayed in Fig. S1–S5 (SI), respectively. Table 3 summarizes the zeta sizes of the fabricated HAp/rGO/Y_2_O_3_ composites. The particle size of the composites is significantly reduced with increasing rGO content in the composite's composition. However, the size also decreases gradually with decreasing Y_2_O_3_ content.

**Table 3:** Zeta size values of the fabricated HAp/rGO/Y_2_O_3_ composites

Sample	AA1	AA2	AA3	AA4	AA5
Size (d. nm) peak1	297.7	249.4	264.2	237.3	211.6
Peak2	4601	4516	5548	1657	974.0
Peak3	000	1197	000	4496	5246

### 3.9. Antimicrobial effectiveness of the fabricated composites

**Fig. 8:**
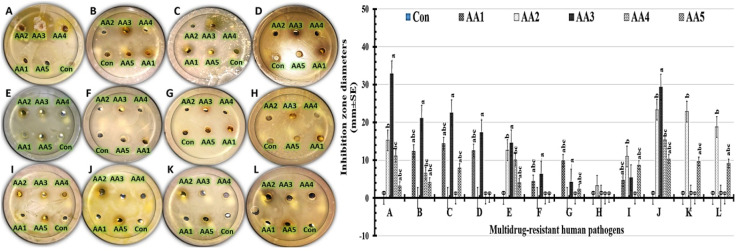
Inhibition zones of the examined nanoformulations (AA1, AA2, AA3, AA4, and AA5) against (A): *Staphylococcus epidermidis*, (B): *Staphylococcus aureus*, (C): *Streptococcus pneumoniae*, (D): *Bacillus cereus*, (E): *Candida albicans*, (F): *Candida krusei*, (G): *Candida tropicalis*, (H): *Candida glabrata*, (I): *Escherichia coli*, (J): *Klebsiella pneumoniae*, (K): *Pseudomonas aeruginosa*, and (L): *Salmonella paratyphi.* From one-way ANOVA, different letters above the bars denote statistically significant differences at *P* ≤ 0.05.

*In vitro* assessment of the antimicrobial performance of the studied nanoformulations:

The size of the inhibitory zone typically serves as an indicator of the antimicrobial agent's efficacy. In our work, the inhibitory zone widths were determined using the well diffusion method to evaluate the antimicrobial properties of the nanoformulations against the tested MDR human pathogens (Fig. 8). The results indicated that each tested formulation's antimicrobial effectiveness was observed at varying degrees against the tested microbes. All pathogens showed the highest antimicrobial activities when the AA3 formula was present (Table 4). The studied formulations generally displayed a variety of antimicrobial activities, ranging from none to highly effective against the tested pathogens. According to statistical data, the AA3 formula showed a wider zone of inhibition against Gram-positive and Gram-negative bacteria than yeast cells. The AA3 formula demonstrated excellent antimicrobial activities in tests with *Staphylococcus epidermidis* (32.87 ± 2.57 mm) and *Klebsiella pneumoniae* (29.35 ± 1.21 mm). It is noteworthy that, as shown in Table 4, the antimicrobial capacity of the AA3 formula against *Candida albicans* (14.54 ± 4.25 mm) was barely noticeable. Similar results have been previously reported, which show that numerous human pathogens cannot propagate when the quantities of graphene oxide and Y_2_O_3_ nanoparticles are increased. Compared to a recent study that employed LC Y_2_O_3_ NPs as an antibacterial agent against Gram-positive bacteria, our AA3 formula showed exceptional inhibitory zones. Small inhibition zones in the range of 11–15 mm were recorded in previous studies, compared to the AA3 formula's range of 20–32 mm.

**Table 4:** Inhibitory zone widths of the tested nanoformulations against multidrug-resistant human pathogens^a^

Multidrug-resistant human pathogens	Inhibition zone diameters (mm ± SD)					
	Con	AA1	AA2**	AA3*	AA4	AA5
*Staphylococcus epidermidis*	0	0	15.23 ± 2.36^ab^	32.87 ± 2.57^a^	11.09 ± 1.09^bc^	3.1 ± 0.09^abc^
*Staphylococcus aureus*	0	12.34 ± 3.58^abc^	0	21.08 ± 2.54^a^	6.54 ± 0.28^bc^	4.1 ± 0.35^abc^
*Streptococcus pneumoniae*	0	14.37 ± 2.04^abc^	0	22.54 ± 4.05^a^	0	7.9 ± 1.15^abc^
*Bacillus cereus*	0	12.52 ± 3.25^abc^	0	17.26 ± 2.25^a^	0	0
*Candida albicans*	0	0	12.63 ± 0.96^ab^	14.54 ± 4.25^a^	10.09 ± 3.54^bc^	4.0 ± 0.22^abc^
*Candida krusei*	0	4.35 ± 0.89^abc^	0	6.35 ± 0.36^a^	0	0
*Candida tropicalis*	0	9.85 ± 2.03^abc^	0	4.25 ± 0.99^a^	0	2.4 ± 0.97^abc^
*Candida glabrata*	0	0	3.21 ± 0.25^ab^	0	0	0
*Escherichia coli*	0	4.69 ± 1.20^abc^	10.98 ± 0.54^ab^	5.36 ± 1.11^a^	0	8.6 ± 2.03^abc^
*Klebsiella pneumoniae*	0	0	23.33 ± 0.33^ab^	29.35 ± 1.21^a^	15.36 ± 1.22^bc^	10.0 ± 2.36^abc^
*Pseudomonas aeruginosa*	0	0	22.85 ± 1.54^ab^	0	0	9.7 ± 1.12^abc^
*Salmonella paratyphi*	0	0	18.78 ± 3.34^ab^	0	0	9.1 ± 2.11^abc^

a A (*) denotes a 5% significant difference. A single star (*) indicates statistically significant differences (*p*-value ≤ 0.05), whereas two stars (**) indicate significant differences (*p*-value > 0.05). Different letters (a–c) indicate statistically significant differences at *p* ≤ 0.05 (one-way ANOVA).

**Fig. 9:**
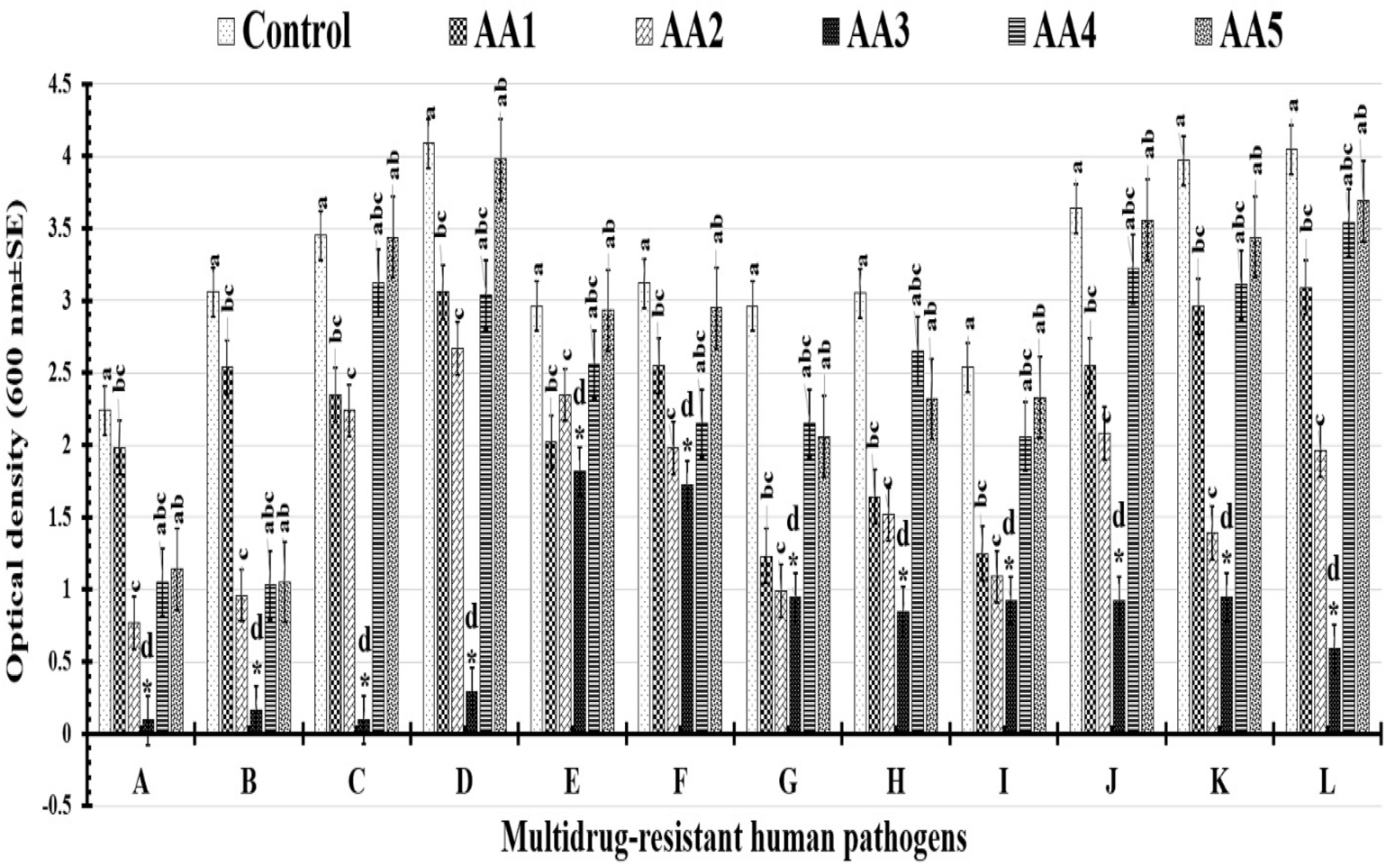
A histogram illustrating the estimated antimicrobial capability of the tested nanoformula against (A): *Staphylococcus epidermidis*, (B): *Staphylococcus aureus*, (C): *Streptococcus pneumoniae*, (D): *Bacillus cereus*, (E): *Candida albicans*, (F): *Candida krusei*, (G): *Candida tropicalis*, (H): *Candida glabrata*, (I): *Escherichia coli*, (J): *Klebsiella pneumoniae*, (K): *Pseudomonas aeruginosa*, and (L): *Salmonella paratyphi* based on turbidity measurements. A single star (*) indicates statistically significant differences (*p*-value ≤ 0.05). Different letters (a–d) indicate statistically significant differences at (*p* ≤ 0.05) (one-way ANOVA).

Compared to the optical densities of the controls, all tested nanoformulations significantly reduced the growth of the tested human pathogens, as shown in Table 5 and Fig. 9. The AA3 formula significantly reduced the turbidity of all pathogens examined, with Gram-positive and Gram-negative bacteria having the largest effects, followed by yeast cells. Notably, the optical density of the control decreased from 3.45 ± 0.69 to 0.094 ± 0.12 when *Streptococcus pneumoniae* was treated with the AA3 formula. Furthermore, the optical density of *Staphylococcus epidermidis* in the control group reduced significantly after treatment with the AA3 formula, from 2.24 ± 0.36 to 0.092 ± 0.01, nearly equaling that of *Streptococcus pneumoniae* treated with the AA3 formula.

**Table 5:** Assessed antimicrobial effectiveness of the tested nanoformulas against MDR human pathogens using turbidity evaluation^a^

Multidrug-resistant human pathogens	Optical density OD (600 nm)					
	Control	AA1	AA2	AA3	AA4	AA5
*Staphylococcus epidermidis*	2.24 ± 0.36^a^	1.98 ± 0.58^bc^	0.77 ± 0.03^c^	0.092 ± 0.01^d^	1.05 ± 0.23^abc^	1.14 ± 0.55^ab^
*Staphylococcus aureus*	3.06 ± 1.24^a^	2.54 ± 0.77^bc^	0.96 ± 0.01^c^	0.16 ± 0.11^d^	1.03 ± 0.58^abc^	1.05 ± 0.11^ab^
*Streptococcus pneumoniae*	3.45 ± 0.69^a^	2.35 ± 0.39^bc^	2.24 ± 0.99^c^	0.094 ± 0.12^d^	3.12 ± 1.22^abc^	3.44 ± 1.55^ab^
*Bacillus cereus*	4.09 ± 1.22^a^	3.06 ± 1.09^bc^	2.67 ± 1.08^c^	0.29 ± 0.19^d^	3.04 ± 1.55^abc^	3.98 ± 2.09^ab^
*Candida albicans*	2.96 ± 1.54^a^	2.02 ± 0.98^bc^	2.35 ± 1.52^c^	1.82 ± 0.51^d^	2.56 ± 1.88^abc^	2.93 ± 1.09^ab^
*Candida krusei*	3.12 ± 1.33^a^	2.55 ± 0.99^bc^	1.98 ± 0.36^c^	1.72 ± 0.97^d^	2.15 ± 1.22^abc^	2.95 ± 1.26 ^ab^
*Candida tropicalis*	2.96 ± 0.57^a^	1.23 ± 0.95^bc^	0.99 ± 0.09^c^	0.95 ± 0.04^d^	2.15 ± 1.56^abc^	2.06 ± 1.33^ab^
*Candida glabrata*	3.05 ± 1.03^a^	1.64 ± 0.09^bc^	1.52 ± 0.79^c^	0.85 ± 0.54^d^	2.65 ± 1.54^abc^	2.32 ± 1.28^ab^
*Escherichia coli*	2.54 ± 0.98^a^	1.25 ± 0.99^bc^	1.09 ± 0.36^c^	0.92 ± 0.09^d^	2.06 ± 0.47^abc^	2.33 ± 1.25^ab^
*Klebsiella pneumoniae*	3.64 ± 0.84^a^	2.55 ± 1.09^bc^	2.08 ± 0.56^c^	0.92 ± 0.34^d^	3.22 ± 0.14^abc^	3.56 ± 2.0^ab^
*Pseudomonas aeruginosa*	3.97 ± 0.97^a^	2.96 ± 0.23^bc^	1.39 ± 0.89^c^	0.95 ± 0.08^d^	3.11 ± 1.33^abc^	3.44 ± 1.55^ab^
*Salmonella paratyphi*	4.05 ± 1.24^a^	3.09 ± 1.22^bc^	1.96 ± 0.94^c^	0.59 ± 0.09^d^	3.54 ± 2.25^abc^	3.69 ± 2.11^ab^

a Different letters (a–d) indicate statistically significant differences at *P* ≤ 0.05 (one-way ANOVA).

**Fig. 10:**
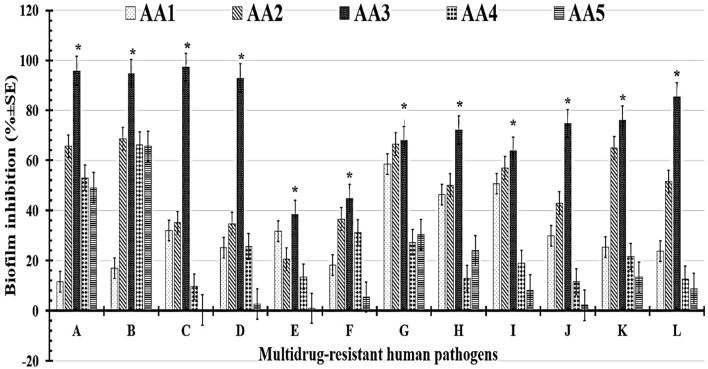
Biofilm inhibition percentages of the tested nanoformulations against (A): *Staphylococcus epidermidis*, (B): *Staphylococcus aureus*, (C): *Streptococcus pneumoniae*, (D): *Bacillus cereus*, (E): *Candida albicans*, (F): *Candida krusei*, (G): *Candida tropicalis*, (H): *Candida glabrata*, (I): *Escherichia coli*, (J): *Klebsiella pneumoniae*, (K): *Pseudomonas aeruginosa*, and (L): *Salmonella paratyphi.* A single star (*) indicates statistically significant differences (*p*-value ≤ 0.05).

The biofilm inhibition rates were calculated *via* antibiofilm analysis to evaluate each formulation's capacity for reducing or preventing the propagation of multidrug-resistant human pathogens. The highest antimicrobial activities, notably against Gram-positive bacteria, were exhibited by the AA3 formula (Table 6 and Fig. 10). The tested AA3 formula showed a promising rate of biofilm inhibition against *Streptococcus pneumoniae* (97.27% ± 5.36%), *Staphylococcus epidermidis* (95.89% ± 2.85%), *Staphylococcus aureus* (94.77% ± 1.24%), and *Bacillus cereus* (92.90% ± 0.95%). Additionally, among Gram-negative bacteria, the tested AA3 formula showed valuable biofilm inhibition rates against *Salmonella paratyphi* (85.43% ± 2.09%), *Pseudomonas aeruginosa* (76.07% ± 0.05%), and *Klebsiella pneumoniae* (74.72% ± 0.52%). The measured biofilm inhibition rates for the AA3 formula against yeast cells, including *Candida glabrata* (72.13% ± 1.14%), *Candida tropicalis* (67.90% ± 0.54%), *Candida krusei* (44.87% ± 1.79%), and *Candida albicans* (38.51% ± 3.51%), were relatively low. In conclusion, yeast cells were more difficult to manage with our tested nanoformula than Gram-positive and Gram-negative bacteria.

**Table 6:** Estimated biofilm inhibition percentages of the tested nanoformulations against MDR human pathogens^a^

Multidrug-resistant human pathogens	Biofilm inhibition (% ± SD)				
	AA1	AA2	AA3	AA4	AA5
*Staphylococcus epidermidis*	11.60 ± 0.71^bc^	65.62 ± 0.95^b^	95.89 ± 2.85^a^	53.12 ± 0.95^c^	49.10 ± 0.71^c^
*Staphylococcus aureus*	16.99 ± 3.46^bc^	68.62 ± 0.74^b^	94.77 ± 1.24^a^	66.33 ± 0.98^c^	65.68 ± 0.62^c^
*Streptococcus pneumoniae*	31.88 ± 4.05^bc^	35.07 ± 2.46^b^	97.27 ± 5.36^a^	9.56 ± 0.52^c^	0.28 ± 0.08^c^
*Bacillus cereus*	25.18 ± 3.37^bc^	34.71 ± 0.88^b^	92.90 ± 0.95^a^	25.67 ± 2.37^c^	2.68 ± 0.94^c^
*Candida albicans*	31.75 ± 0.67^bc^	20.60 ± 0.81^b^	38.51 ± 3.51^a^	13.51 ± 3.51^c^	1.01 ± 0.35^c^
*Candida krusei*	18.26 ± 0.92^bc^	36.53 ± 0.84^b^	44.87 ± 1.79^a^	31.08 ± 0.97^c^	5.44 ± 0.87^c^
*Candida tropicalis*	58.44 ± 0.59^bc^	66.55 ± 4.05^b^	67.90 ± 0.54^a^	27.36 ± 4.86^c^	30.40 ± 0.54^c^
*Candida glabrata*	46.22 ± 0.95^bc^	50.16 ± 3.93^b^	72.13 ± 1.14^a^	13.11 ± 0.47^c^	23.93 ± 0.44^c^
*Escherichia coli*	50.78 ± 0.74^bc^	57.08 ± 0.66^b^	63.77 ± 0.95^a^	18.89 ± 0.76^c^	8.26 ± 0.77^c^
*Klebsiella pneumoniae*	29.94 ± 0.55^bc^	42.85 ± 0.71^b^	74.72 ± 0.52^a^	11.53 ± 0.84^c^	2.19 ± 0.78^c^
*Pseudomonas aeruginosa*	25.44 ± 0.86^bc^	64.98 ± 0.7^b^	76.07 ± 0.05^a^	21.66 ± 2.46^c^	13.35 ± 0.12^c^
*Salmonella paratyphi*	23.70 ± 3.73^bc^	51.60 ± 4.93^b^	85.43 ± 2.09^a^	12.59 ± 2.59^c^	8.88 ± 0.28^c^

a Different letters (a–c) indicate statistically significant differences at *P* ≤ 0.05 (one-way ANOVA).

Additionally, multiple dosages of the AA3 nanoformula (20, 40, 60, 80, 100, 120, 140, 160, 180, 200, and 220 µg mL^−1^) were used to determine the minimum inhibitory dose against multidrug-resistant human pathogens. As shown in Table 7, Gram-positive bacteria exhibited a range of MIC values from 80 to 100 µg mL^−1^, whereas Gram-negative bacteria exhibited an observed MIC range from 100 to 120 µg mL^−1^. Additionally, the MIC range for yeast cells was between 120 and 140 µg mL^−1^. The AA3 formula's lowest concentration at which no recognizable microbial life could be detected was identified as the MBC/MFC (Table 7). The AA3 formula had a bactericidal impact against Gram-positive bacteria at a concentration of 120 µg mL^−1^, which is lower than that found to be effective against Gram-negative bacteria (140–160 µg mL^−1^). The MFC value against the studied yeast cell was 180–200 µg mL^−1^.

**Table 7:** Inhibitory doses of the AA3 nanoformula (20, 40, 60, 80, 100, 120, 140, 160, 180, 200, and 220 µg mL^−1^) against MDR human pathogens using the microdilution method. (MIC): minimum inhibitory concentration, (MBC): minimal bactericidal concentration, and (MFC): minimal fungicidal concentration

Multidrug-resistant human pathogens	MIC	MBC	MFC
*Staphylococcus epidermidis*	80	120	
*Staphylococcus aureus*	100	120	
*Streptococcus pneumoniae*	80	120	
*Bacillus cereus*	100	120	
*Candida albicans*	120		180
*Candida krusei*	140		200
*Candida tropicalis*	120		180
*Candida glabrata*	120		200
*Escherichia coli*	100	140	
*Klebsiella pneumoniae*	120	160	
*Pseudomonas aeruginosa*	100	140	
*Salmonella paratyphi*	120	160	

According to Radwan *et al.*,^25^ nanographene oxide sheets are more effective against Gram-positive bacteria. These results confirmed our conclusions. Gram-positive and Gram-negative bacteria have been successfully destroyed by nanographene oxide (GO) sheets. Due to its highly oxygenated surface, which also has functional groups such as hydroxyl, epoxy, and carbonyl, rGO can easily swell and spread in a solution. rGO can be dispersed as a single sheet because of the electrostatic repulsion and solvation that oxygen species' negative charge provides.^25^

Several mechanisms have been proposed to be responsible for its antimicrobial effects. Reactive groups in GO, which target lipids, as well as DNA, RNA, and cell proteins, suggest that GO and cell walls have a powerful connection.^25^ The microbe's cytoplasmic membrane is damaged, specifically when lipids are attacked because of decreased membrane fluidity, altered membrane properties, and disrupted membrane-bound proteins.^26^ Another theory posits that GO sheets capture pathogen cells, damage cells with their sharp edges, and transfer electrons from the membranes of the pathogens to GO.^27^ Gram-positive bacteria have shown the greatest sensitivity to GO. The differences between Gram-positive and *Gram-negative* bacteria's cell shapes, as well as the propensity of Gram-positive bacteria to cluster, may also be to blame. Compared to Gram-negative bacteria, which are typically found as single or paired cells, Gram-positive bacteria are more likely to form clusters that increase GO exposure. Gram-positive bacteria, as a result, routinely exhibit greater sensitivity to GO. Gram-positive bacteria contain several peptidoglycan layers in their cell walls, whereas the cell walls of Gram-negative bacteria are formed of an exterior porous layer of lipopolysaccharide. According to some theories, this leads to an increase in the contact of Gram-positive bacteria with GO *via* electrostatic or hydrogen bonding.^25^ The specifics of how the generated nanomaterials, such as Y_2_O_3_, interact with various pathogen strains are still unknown to researchers. However, several factors, such as the nanoformula's fragmentation and the particles' shapes and sizes (surface area), have been proposed to explain the mode of action of the nanoparticles as an antimicrobial agent. Reactive oxygen species (ROS) production and the electrostatic interaction of the nanoformula with the pathogen's cell wall also influence its antimicrobial abilities. When the examined pathogens were exposed to the nanoformulae, the formation of ROS, like hydroxyl (OH), hydrogen peroxide (H_2_O_2_), and superoxide anions (O_2_), was increased. The pathogen's cell membrane and respiratory system are both impacted by the ROS produced, which finally destroys the microbe.

### 3.10. Cell culture tests of the fabricated composites

**Fig. 11:**
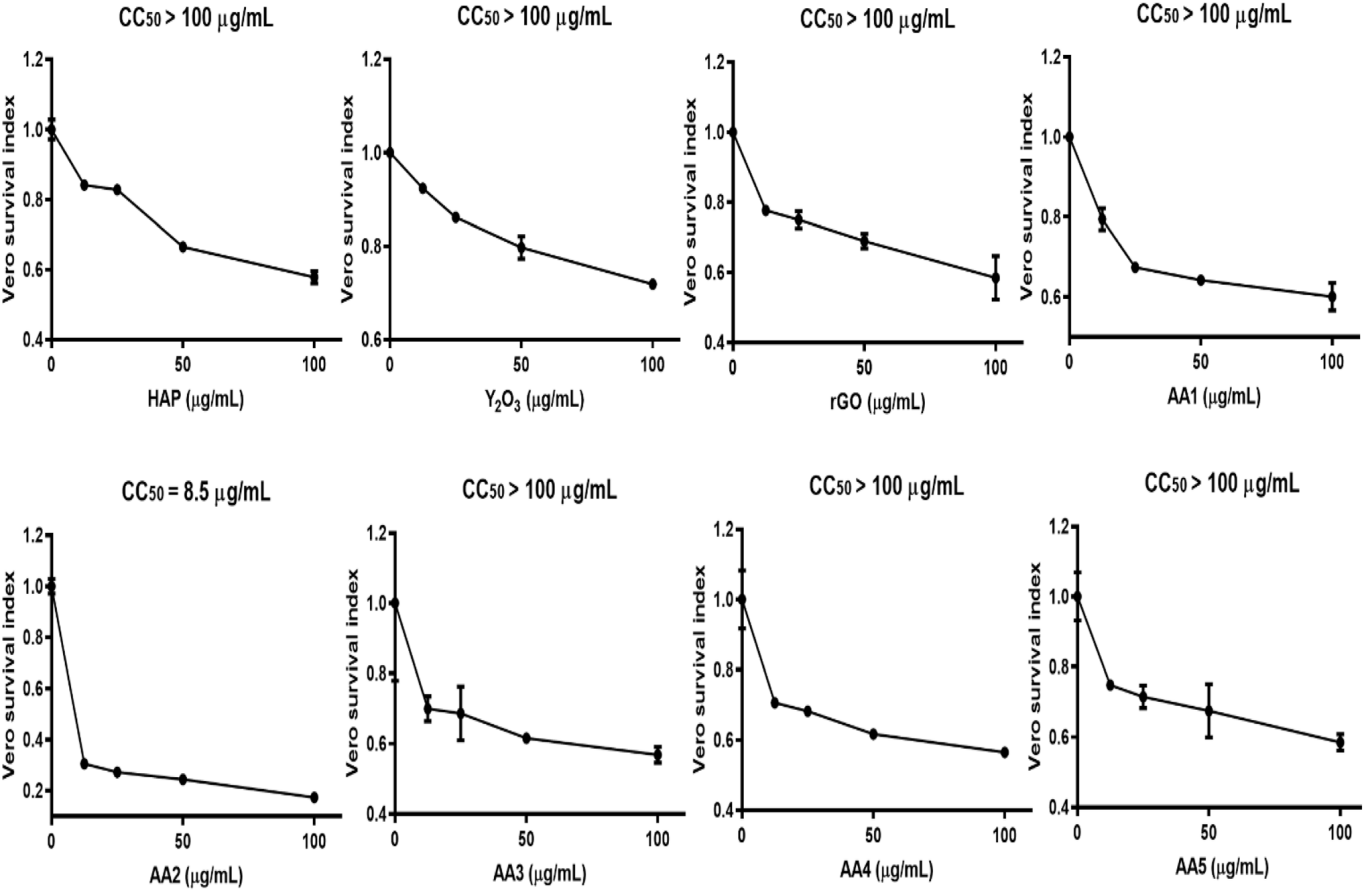
CC_50_ values of HAp, rGO, and Y_2_O_3_ and different HAp/rGO/Y_2_O_3_ composites obtained using the *Vero* cell line.

**Fig. 12:**
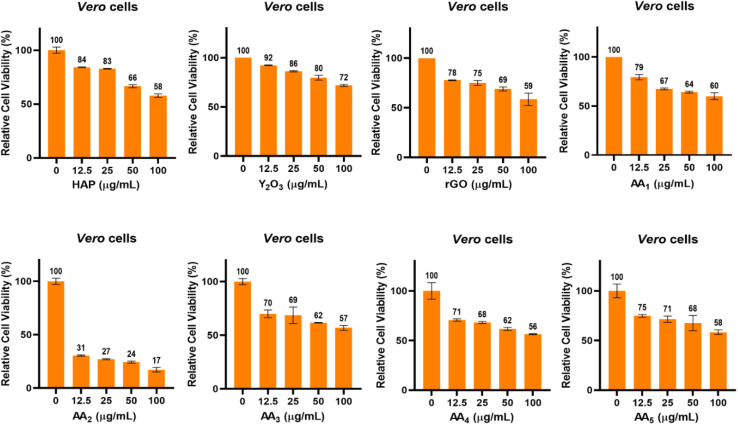
Cell viability (%) test by the MTT assay using the *Vero c*ell line for HAp, rGO, and Y_2_O_3_ and different HAp/rGO/Y_2_O_3_ composites.

The MTT colorimetric assay was performed to validate the biocompatibility of the tested materials and their composites and their potential for use in biomedical applications. As shown in Fig. 11, the results revealed that CC_50_ values for *Vero* cells exceeded a concentration of 100 µg mL^−1^ for HAp, Y_2_O_3_, rGO and composites AA1, AA2, AA3, AA4, and AA5, while the CC_50_ value of AA2 was detected at a concentration of 8.5 µg mL^−1^. Meanwhile, for all tested materials and composites, the cellular viability of *Vero* cells decreased gradually with increasing material concentration ranging from 12.5 to 100 µg mL^−1^ (Fig. 12). HAp, Y_2_O_3_ and rGO showed favorable biocompatibility results, maintaining cellular viability in the range from 58% to 72% at the highest concentration of 100 µg mL^−1^.^26,27^

Moreover, the cellular viability results of composites AA1, AA2, AA3, AA4, and AA5 indicated relatively good biocompatibility profiles with cellular viability ranging from 56% to 60% at a concentration of 100 µg mL^−1^. In contrast, AA2 demonstrated the highest degree of cytotoxicity, reducing cell viability to 17% at the same concentration. It is suggested that the composition of the AA2 composite may induce a possible synergistic cytotoxic effect on *Vero* cells. This may be attributed to the enhanced generation of reactive oxygen species (ROS) or membrane damage caused by the composite.^33^

## 4 Conclusions

In this study, novel nanocomposites based on hydroxyapatite (HAp) reinforced with reduced graphene oxide (rGO) and yttrium oxide (Y_2_O_3_) were successfully synthesized *via* a sol–gel method, and their structural, morphological, optical, and functional properties were thoroughly characterized, with a particular focus on their potential applications in cancer therapy. XRD analysis confirmed the formation of well-crystallized hydroxyapatite with characteristic diffraction peaks, while the incorporation of rGO and Y_2_O_3_ introduced slight peak broadening and shifts, indicative of lattice strain and nanocomposite formation. The crystallite size decreased with the inclusion of rGO and Y_2_O_3_, supporting the formation of finer nanostructures, suitable for biomedical interfaces. FT-IR spectra exhibited the presence of characteristic phosphate (PO_4_^3−^), hydroxyl (OH^−^), and carbonate groups, with additional peaks associated with rGO and Y_2_O_3_, confirming their successful incorporation into the HAp matrix. The shifts in vibrational bands further suggested strong interfacial interactions among the components. SEM analysis revealed that the prepared composites exhibited a granular morphology with quasi-spherical particles and an average particle size of approximately 380 nm. The morphology was homogeneous and compact, which is favorable for both mechanical integrity and biological integration. Optical property analysis using UV/vis and *Tauc* plots demonstrated that the addition of rGO and Y_2_O_3_ led to a notable reduction in the optical bandgap (3.92 eV, 3.82 eV, 3.08 eV, 2.72 eV, and 2.66 eV for AA1, AA2, AA3, AA4, and AA5, respectively). This bandgap narrowing is beneficial for enhancing photothermal response and energy absorption characteristics, which are critical for targeted cancer therapies involving light-mediated mechanisms. Finally, the combined inclusion of rGO (imparting excellent electron transport and photothermal behavior) and Y_2_O_3_ (providing reactive oxygen species generation and improved mechanical stability) into the HAp matrix resulted in multifunctional bioceramic nanocomposites with tailored physicochemical and optical properties. These findings suggest that the HAp/rGO/Y_2_O_3_ nanocomposites hold strong potential for use in bone tumor therapeutic systems, enabling simultaneous bone regeneration, drug delivery, and localized cancer treatment *via* photothermal or oxidative mechanisms.

## Ethical approval

This article does not contain any studies with animals performed by any of the authors.

## Author contributions

Ahmed I. Ali, Asmaa Elawadly: synthesis and characterization of samples. Merna H. Emam methodology, formal analysis, data curation and cell culture experiments. Shahira H. EL-Moslamy, Asmaa M. Elzayat, and E. M. Abdelrazek: resources, visualization, validation, and antimicrobial testing experiments. Mohammed Sallah: supervision. Elbadawy A. Kamoun, Ahmed I. Ali, and Jong Yeog Son: supervision, writing – review & editing, writing – original draft, validation, supervision, and project administration. All authors approved the current final version for submission.

## Conflicts of interest

The authors declare that they have no known competing financial interests or personal relationships that could have appeared to influence the work reported in this paper.

## Supplementary Material

RA-016-D5RA08618C-s001

## Data Availability

All data generated or analyzed during this study are included in this submitted manuscript. Supplementary information (SI) is available. See DOI: https://doi.org/10.1039/d5ra08618c.
