# Supplementary material for: Multifunctional rGO/Y2O3@hydroxyapatite bioceramics: structural, optical, and biomedical properties
Source: RSC Adv. 2026 Jan 23;16(6):5264–80. doi: 10.1039/d5ra08618c (PMC12829543; doi:10.1039/d5ra08618c)
Supplement: RA-016-D5RA08618C-s001 [file RA-016-D5RA08618C-s001.pdf]

Supplementary Information (SI) for RSC Advances.
This journal is © The Royal Society of Chemistry 2026

Supplementary Information

Multifunctional rGO/Y_2O_3@Hydroxyapatite Bioceramics: Structural, Optical, and Biomedical Properties

Elbadawy A. Kamoun[1,2*], Asmaa Elawadly[2], Merna H. Emam[3], Shahira H. EL-Moslamy[4], Asmaa M. Elzayat[2], E. M. Abdelrazek[2], Mohammed Sallah[5], Jong Yeog Son[6*], and Ahmed I. Ali[7,8*]

[1]*Department of Chemistry, College of Science, King Faisal University, Al-Ahsa 31982, Saudi Arabia.*
[2]*Physics Department, Faculty of Science, Mansoura University, Mansoura 35516, Egypt.*
[3]*Nanotechnology Research Center (NTRC), The British University in Egypt, El-Shorouk City, Suez Desert Road, P.O. Box 43, Cairo 11837, Egypt.*
[4]*Bioprocess Development Department (BID), Genetic Engineering and Biotechnology Research Institute (GEBRI), City of Scientific Research and Technological Applications (SRTA-City), New Borg El-Arab City, Alexandria 21934, Egypt.*
[5]*Department of Physics, College of Sciences, University of Bisha, P.O. Box 344, Bisha 61922, Saudi Arabia.*
[6]*Department of Applied Physics and Institute of Natural Sciences, College of Applied Science, Kyung Hee University, Suwon 446-701, Republic of Korea*
[7]*Basic Science Department, Faculty of Technology and Education, Helwan University, Saray-El Qoupa, El Sawah St.,11281 Cairo, Egypt.*
[8]*Department of Mechanical Engineering (Integrated Engineering Program), Kyung Hee University, 1732 Deogyeong-daero, Yongin, Gyeonggi, 17104, South Korea.*

**Corresponding authors: (E. A. Kamoun: ekamoun@kfu.edu.sa, badawykamoun@yahoo.com, Tel: +201283320302, (Jong Yeog Son; jyson@khu.ac.kr) (Ahmed I. Ali: ahmed_ali_2010@techedu.helwan.edu.eg, ahmedalikh2024@khu.ac.kr).*

- *Particle size analysis of rGO/Y_2O_3@Hap composites*

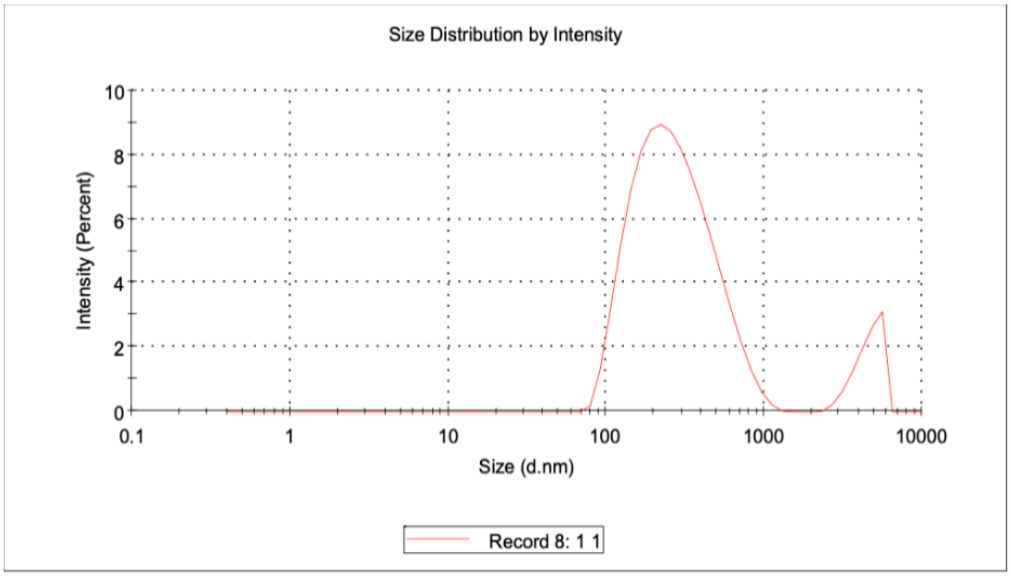

Fig. S1 Zeta size of composite coded AA1.

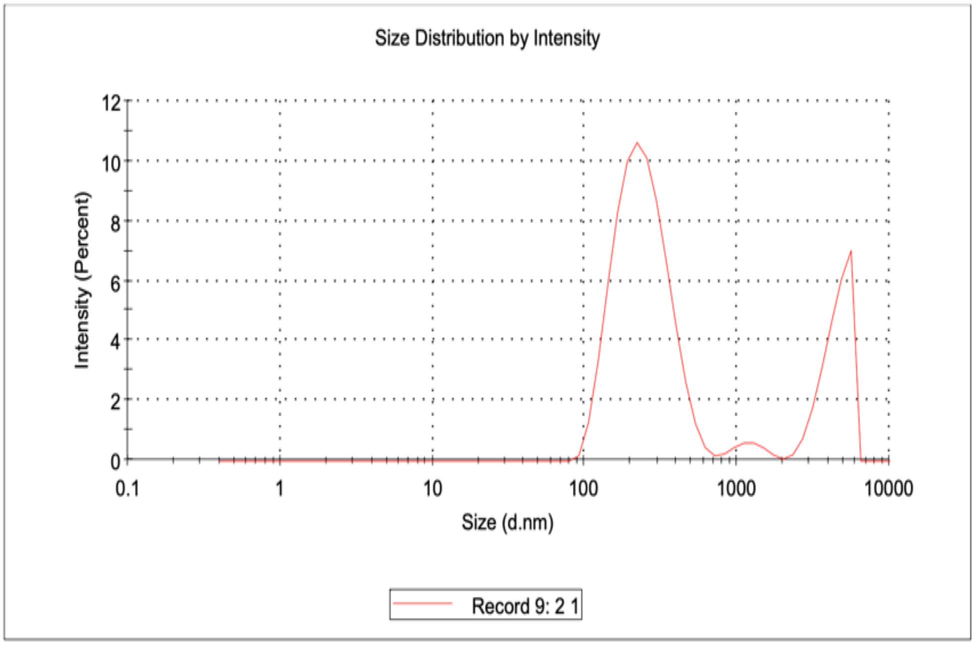

Fig. S2 Zeta size to of composite coded AA2.

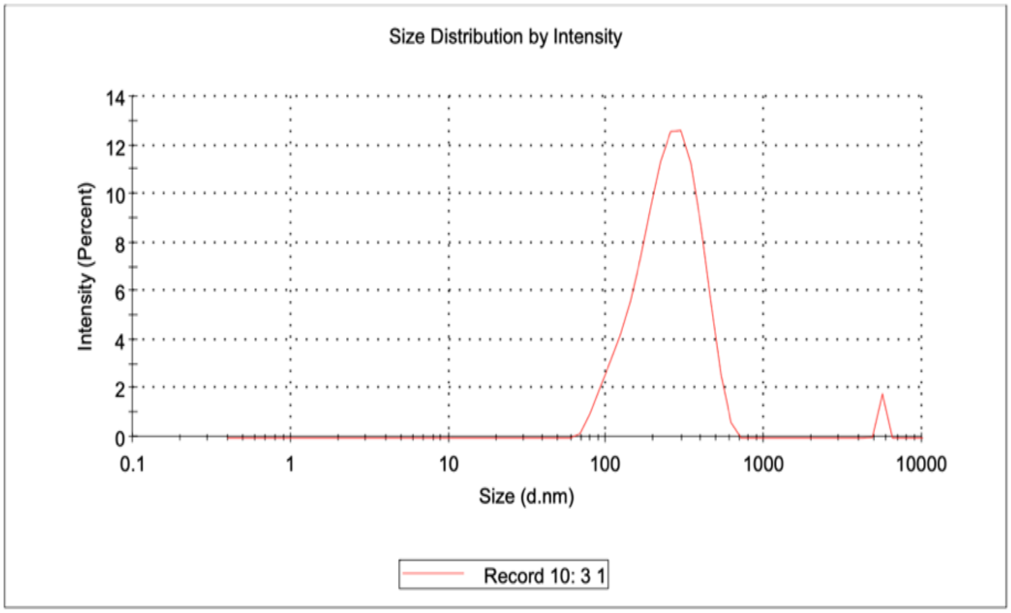

Fig. S3 Zeta size to of composite coded AA3.

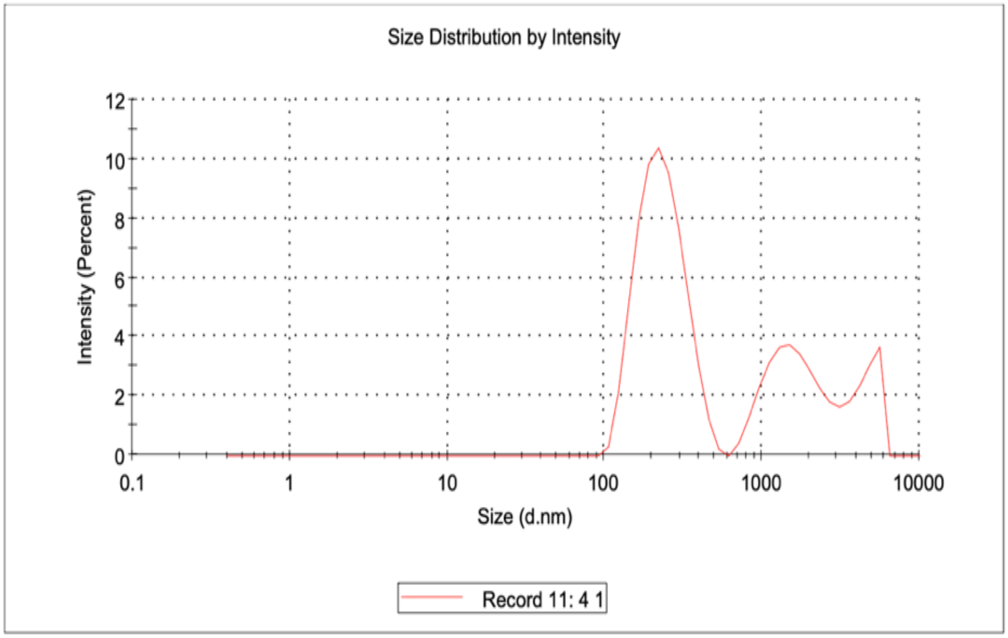

Fig. S4 Zeta size to of composite coded AA4.

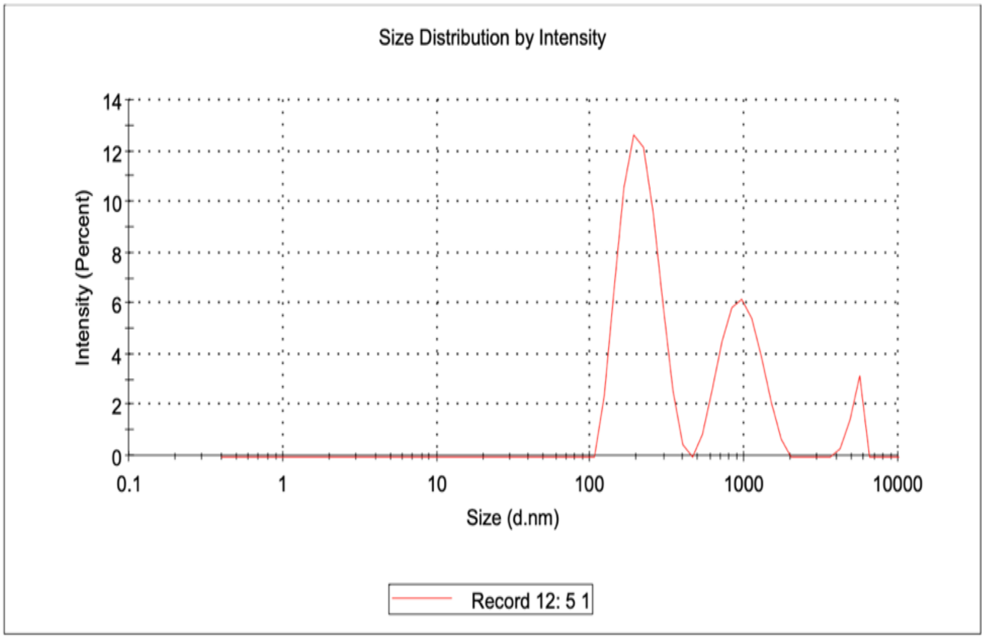

Fig. S5 Zeta size to of composite coded AA5.
